# Enhancement of mechanical and electrical properties of continuous-fiber-reinforced epoxy composites with stacked graphene

**DOI:** 10.3762/bjnano.8.191

**Published:** 2017-09-12

**Authors:** Naum Naveh, Olga Shepelev, Samuel Kenig

**Affiliations:** 1 Shenkar College of Engineering and Design, 12 Anna Frank St., Ramat Gan 5252626, Israel; 2 Israel Plastics and Rubber Center, Technion City, Haifa 3200004, Israel

**Keywords:** composite, exfoliation, graphene, surface-active agents (SAAs), thermo-mechanical properties

## Abstract

Impregnation of expandable graphite (EG) after thermal treatment with an epoxy resin containing surface-active agents (SAAs) enhanced the intercalation of epoxy monomer between EG layers and led to further exfoliation of the graphite, resulting in stacks of few graphene layers, so-called “stacked” graphene (SG). This process enabled electrical conductivity of cured epoxy/SG composites at lower percolation thresholds, and improved thermo-mechanical properties were measured with either Kevlar, carbon or glass-fiber-reinforced composites. Several compositions with SAA-modified SG led to higher dynamic moduli especially at high temperatures, reflecting the better wetting ability of the modified nanoparticles. The hydrophilic/hydrophobic nature of the SAA dictates the surface energy balance. More hydrophilic SAAs promoted localization of the SG at the Kevlar/epoxy interface, and morphology seems to be driven by thermodynamics, rather than the kinetic effect of viscosity. This effect was less obvious with carbon or glass fibers, due to the lower surface energy of the carbon fibers or some incompatibility with the glass-fiber sizing. Proper choice of the surfactant and fine-tuning of the crosslink density at the interphase may provide further enhancements in thermo-mechanical behavior.

## Introduction

Carbon nanotubes (CNTs) have been suggested as an efficient conductive filler because of the outstanding electrical properties and the high aspect ratio. CNT-modified carbon epoxy composites have been studied, where the CNTs are either dispersed in the matrix and/or grafted on the carbon fibers [1–2]. However, the cost of CNTs limits intensive industrial applications. Other treatments have been attempted, among these, oxidation of the carbon fibers, plasma treatment, radiation, modification with rubber, silica, carbon or other nanoparticles, showing interesting enhancements in interlaminar shear strength (ILSS), fracture toughness, fatigue life and related properties [2–6].

Graphite nanoplatelets (GNPs) or stacked graphene (SG) have been developed as a low-cost conductive alternative [7]. GNPs can be produced by intercalation of the graphitic layers with an appropriate agent followed by exfoliation of the graphite flakes. Exfoliation is obtained by rapid heating resulting in conversion of the intercalant to a gas phase forcing the adjacent graphene layers to separate. Consequently, formation of worm-like accordion structured materials takes place with partially separated graphite sheets, i.e., expanded graphite (EG), characterized by a high expansion ratio of 100–400 cm^3^/g. For instance, the increase to about 200 cm^3^/g caused a thickness increase of about 80–100 times by a thermal shock at 600 °C [8]. Sonication in an acetone bath allowed for further exfoliation and separation of the loosely connected graphite nanosheets.

Further size reduction of these large (several micrometers long) structures produces fine particles consisting of a few layers of graphene loosely joined together, i.e., SG or GNP, also called few layered graphene (FLG). The exfoliation volume is governed by the structure of the starting expandable graphite, exfoliation temperature and heat rate [9]. The largest increase in volume to 300 cm^3^/g at 900 °C was obtained by exfoliating graphite treated by a mixture of H_2_SO_4_/HNO_3_/KMnO_4_ at a ratio of 1:9:3:0.44 over an immersing time of 150 min in formic acid [10]. Intercalation with 98% HNO_3_ followed by hydrolysis resulted in the the formation of graphite nitrate with negligible damage to the sp^2^ graphite lattice. An interlayer distance of 0.336 nm was measured. Nevertheless, numerous multi-pores ranging from 2 to 10 nm were also detected. The acid and hydroxy groups on the multi-pore walls promote the interaction of EG with organic compounds, more specifically, with monomers before curing and with polymer segments after curing, thus contributing to the mechanical properties and percolation threshold reduction of conductive polymer/graphite nanocomposites [11–12].

Graphite intercalation compounds (GIC) may provide a plausible high-yield source for polymer nanocomposites [8]. However, most of the methods described in the literature either require expensive chemicals and/or are characterized by low yields of the final material, and therefore are hardly applicable for mass production.

Low percolation thresholds have been demonstrated in polymer compositions with nanosized fillers. The high aspect ratio and large surface area of graphene, along with the high electrical conductivity, promote percolation thresholds much lower than with metallic powders, carbon fibers or carbon black [13].

Epoxy resins are used as a matrix in high-performance composite materials for aerospace structures, coatings and adhesives for a variety of applications. Epoxy systems combined with reinforcing fibers provide composites with high strength and stiffness, ease of molding complex shapes and environmental resistance at low densities. The properties of epoxy systems can be varied as a function of the molecular weight or the functionality of the hardener constituent, by variations in processing conditions or by changing the ratio between hardener and monomer. Incorporation of functional silanes can improve the dispersibility of fillers in an epoxy system and increase the mechanical properties of the cured resin. The combination of SG and epoxy fiber composites was hardly studied. Nevertheless, it may offer special properties by reinforcing the epoxy matrix and providing higher electrical conductivities depending on the localization of the SG. Consequently, the present investigation deals with epoxy-based conductive compositions containing treated stacked graphene and continuous fabric reinforcement based on carbon fibers, Aramid and glass fibers.

## Experimental

### Materials

Diglycidyl ether of bisphenol A (DGEBA) (DER 331, Dow Chemical Company) with epoxy equivalent weight (EEW) 182–192; triethylenetetramine (TETA) hardener, amine equivalent weight 24 (LEUNA-Harze GmbH); graphite intercalated compound (GIC) of 30–50 mesh, 3.1% sulfur (3772, Anthracite Industries, Inc. a subsidiary of Asbury Carbons); surface-active agents (SAAs) for SG treatment used in this study: polyether polyol (*M*_W_ = 4000, hydroxyl number: 28 mg KOH/g, (Grade 4200 from Bayer)), octylphenol ethoxylate (Triton X-100, HLB = 13.5, and Triton X-15, HLB = 4.9), as well as 3-methacryloxypropyltrimethoxysilane (MEMO) and (3-glycidyloxypropyl)trimethoxysilane (GLYMO) (Sigma-Aldrich); fabrics used: 3k carbon fiber of plain weave, 200 g/m^2^ (Primetex ZB type 43199 from Hexcel), Kevlar of plain weave, 450 g/m^2^ (Type 745) and fiberglass fabric of plain weave, 300 g/m^2^.

### Preparation of expanded graphite (EG)

The commercial sulfuric acid intercalated expandable graphite (GIC 3772) was subjected to thermal shock at 600–620 °C in a preheated muffle furnace in air, with further heating of the expanded material for 15–20 min to perform homogeneous exfoliation of the graphite sheets into stacked graphene (SG) consisting of layered graphene structures.

### Preparation of epoxy compositions

The properties of epoxy-based compositions with various concentrations of EG incorporated by different techniques were investigated. The principle method comprised the following steps: impregnation at elevated temperatures of EG in epoxy resin premixed with SAAs, further size reduction of EG worm-like particles using intensive mixing, and degassing of the paste-like epoxy/SG compositions in vacuum to remove the entrapped air. Two nonionic surfactants (Triton X100 and X-15) with different hydrophilic/lyophilic balance (HLB) were used in order to evaluate the thermodynamic effect on the localization of the SG. Hardener TETA was added to the mixture before specimens casting.

The basic formulation (by weight) was as follows: epoxy: 100 parts; SAA: 1 pph; expanded graphite (EG): 2.5 pph (or 2.1 wt %); hardener TETA: 14.1 pph.

### Fabrication of composite materials

Composite laminates were prepared using carbon fiber (CF) fabric, Kevlar fabric alone or a combination of CF/Kevlar combinations, and fiberglass (FG) fabrics. CF was used as received, and Kevlar was cleaned by dipping in isopropyl alcohol (IPA) with further drying.

FG was used as received or modified by silanes and surfactants. FG specimens (150 × 150 mm) were dipped in solutions of MEMO and GLYMO silanes in IPA/water. The silane/IPA/water ratio was 2:90:10. Acetic acid (2 g) was added to adjust the pH value of the solution to 5.0. Wet specimens were rinsed with IPA, dried at room temperature and finally cured at 120 °C for 30 min.

Specimens of FG fabrics were heat-treated at 600 °C for 2 h to burn out the sizing material and dried at 150 °C just before dipping into a variety of treatment solutions. Solutions of (3-aminopropyl)triethoxysilane (AMEO), Triton X-15 and TETA in IPA were used as modifiers of the FG surface.

Laminates were fabricated by the hand lay-up technique using brush and roller to apply the matrix composition on the fabric plies. The samples were designed to provide a thickness of 2.5–3.0 mm. The fiber volume fraction was controlled by applying pressure to the laminates to a predetermined thickness. The samples were cured under pressure for two days at ambient temperature, and after that post-cured at 80 °C for 4 h and at 120 °C for 2 h.

### Characterization

The thermo-mechanical properties of the compositions were measured at 1 Hz according to ASTM D 4065 using a DMA-Dynamic Mechanical Analysis (Q800 TA Instruments). Flexural testing followed ASTM D790 (3 point bending) at 1.3 mm/min. Electrical resistivity, both surface and volume, was determined with an electrometer (Keithley 6517D). Typically, the characterization comprised five specimens with standard deviations of 5–8%. Morphology of the composite materials was characterized with high-resolution scanning electron microscopy (Zeiss). Specimens were cut from samples frozen in liquid nitrogen.

## Results and Discussion

### Composite materials based on Kevlar

**Figure 1:**
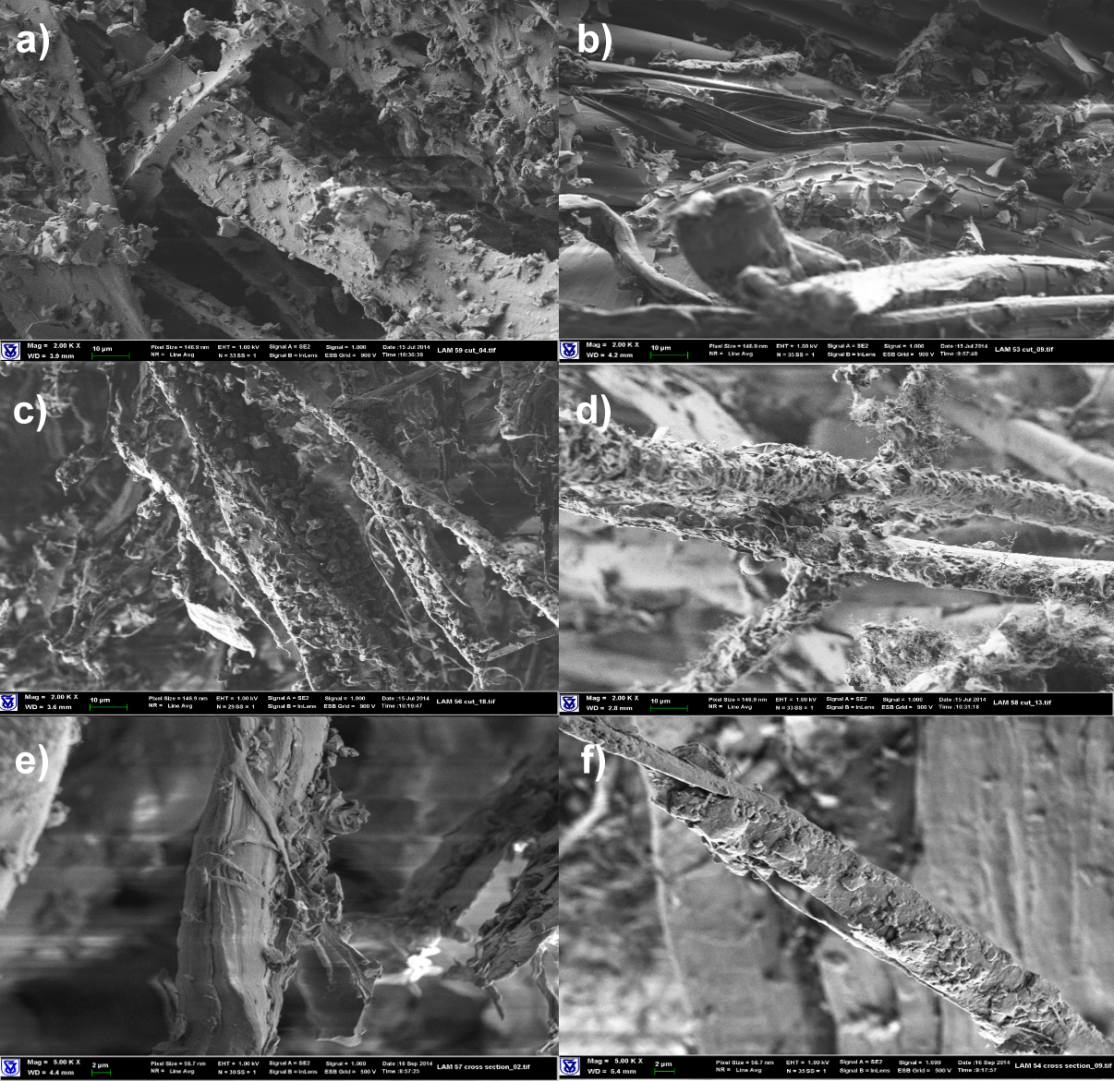
SEM images of composite materials based on Kevlar fabric (magnification: 2000×). (a) Neat Epoxy matrix, (b) epoxy/SG (2.5 pph) matrix, (c) epoxy/Triton X-100 (1 pph)/SG (2.5 pph) matrix, (d) epoxy/MEMO (1 pph)/SG (2.5 pph) matrix, (e) epoxy/Triton X-15 (1 pph)/SG (2.5 pph) matrix, (f) epoxy/GLYMO (1 pph)/SG (2.5 pph) matrix.

The electrical, mechanical and thermo-mechanical properties of composite materials prepared with Kevlar fabrics are exhibited in Table S1 (Supporting Information File 1). Laminated composites based on Kevlar fabrics with epoxy/SG matrix demonstrated good electrical properties, despite the insulating nature of the fabric. Enhanced rigidity in three-point bending and increased DMA storage moduli were achieved for compositions where EG was impregnated with epoxy resin modified with the surfactants Triton X-100, Triton X-15 or MEMO. These results are in good correlation with the changes in morphology of the composite materials determined by SEM. It can be seen in Figure 1 that the morphology of the composite materials changes depending on the composition of the matrix. Combination of epoxy resin with Triton X-100 and MEMO provides improved wetting of the Kevlar fabric with the matrix compound. Kevlar filaments are covered with adhered particles of SAA/SG, while laminates prepared with the same neat epoxy or unmodified epoxy/SG exhibit bare surfaces of the fabric. Triton X-100 and MEMO have lower viscosities than Triton X-15 and Polyol 4200, this supports their contribution to better wetting.

For compositions in which epoxy resin was modified by hydrophobic Triton X-15 or GLYMO the fiber coverage is less pronounced. Some uncovered sections of Kevlar filaments can be observed in Figure 1e,f. This is in spite of the very low viscosity of GLYMO. The hydrophilic/hydrophobic nature of the SAA dictates the surface energy balance, such that more hydrophilicity (thus, higher surface energies) drives the treated nanoparticles to the fiber/matrix interface, due to the higher surface energy of the Kevlar fibers. The morphology is thermodynamically driven, and the proper choice of the surfactant controls the localization of SG at the interface. However, a higher concentration of SG on the fibers does not translate into better mechanical properties. Composites with Polyol 4200 or Triton X-15 show the highest flexural strengths in spite of the larger viscosities and the, supposedly, unfavorable surface energy. We may speculate on the weak contribution of Triton X-100 and MEMO to strength, compared to Triton X-15 and Polyol 4200. The effect of better wetting is second to the more significant weakening effect of the interphase by the low molecular weight of the SAAs. Thus, to enhance the properties of the interface wetting is not sufficient. It might also be the case that more hydrophobic SAAs interact more strongly with SG, allowing for a better local dispersion. Further work is required to unveil the contributions of both thermodynamic and dynamic effects, but, in general, one can state that stronger interfaces are reflected in the higher flexural strength of the composites.

### Composite materials based on carbon fabrics

**Figure 2:**
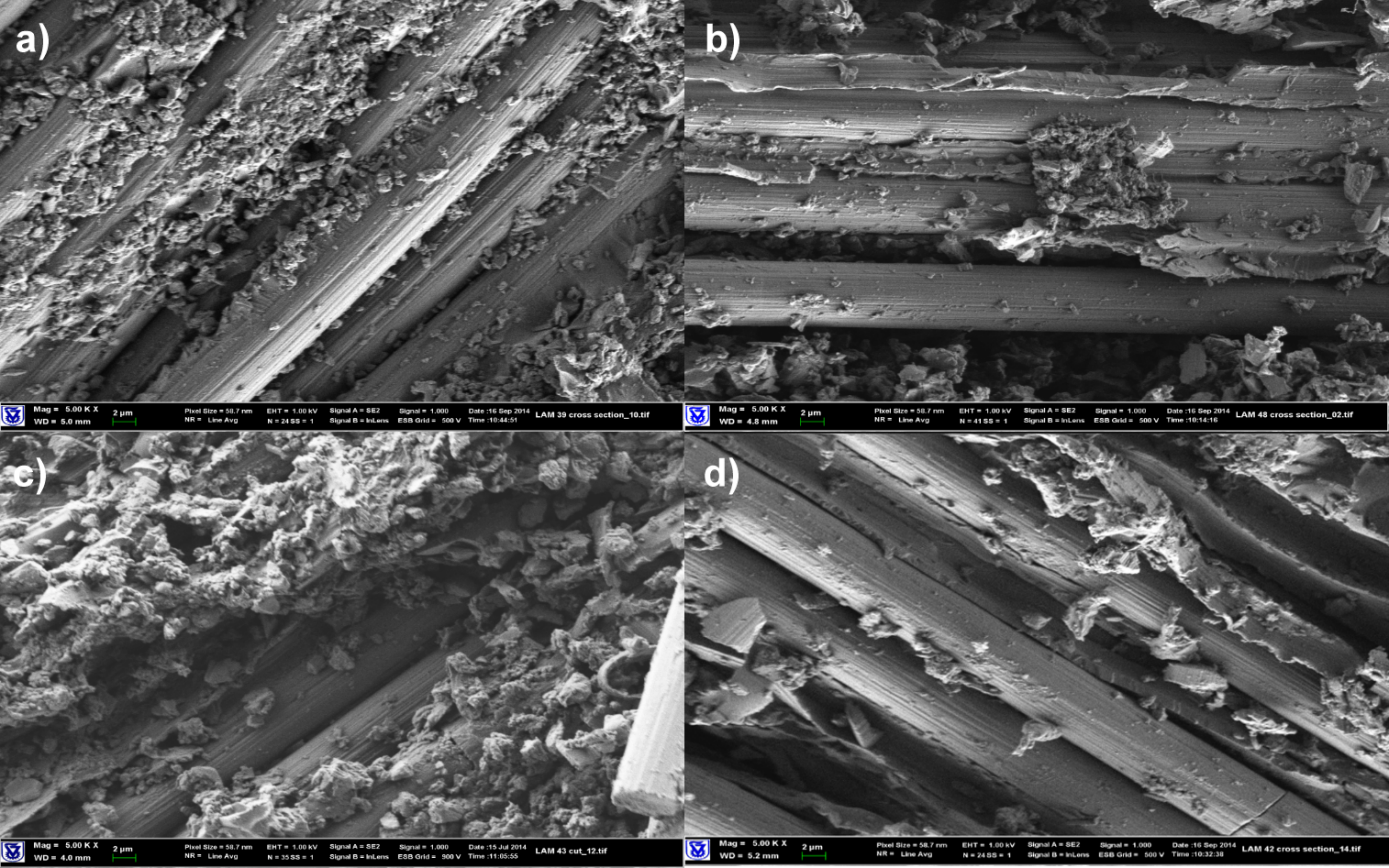
SEM images of composite materials based on carbon fabrics (magnification 5000×). (a) Neat epoxy matrix, (b) epoxy/MEMO (1 pph)/SG (2.5 pph) matrix, (c) epoxy/Triton X-100 (1 pph)/SG (2.5 pph) matrix, (d) epoxy/Triton X-15 (1 pph)/2.5 pph SG matrix.

SEM images of composite materials based on carbon fabrics in Figure 2 did not reveal distinctive differences in morphology. Due to the lower surface energy of the carbon fibers, compared to Kevlar, the driving force for localization of the nanoparticles at the fiber/matrix interface is weaker, and less coverage of the fabric is obtained. The effect of SAAs on the flexural strength is either negligible or minor but negative, and SG agglomeration seen in Figure 2 is supporting these findings.

All laminated composites manifested increased DMA storage moduli. Composites with modified epoxy/SG matrices demonstrated a significant increase of storage moduli at elevated temperatures, and higher Young’s moduli (Table S2, Supporting Information File 1). The distributed SG within the epoxy matrix contributes to increase in modulus, a 10% increase is seen in the range of 25–100 °C, and a significant 60% increase is exhibited at 120 °C with either SG treated with Triton X-100 or MEMO. Further exfoliation of SG by SAAs leads to higher moduli, and in the case of Polyol 4200 the modulus is almost doubled compared to the neat matrix. Again, the higher viscosity of Polyol 4200 is not detrimental to its role in enhancing the properties, and the good interaction with SG is due to a low surface energy.

The surface resistivity of the composite materials with epoxy/SG matrix systems dropped by 7–8 orders of magnitude, but the volume resistivity remained at the level of carbon fabrics/neat epoxy composite. Resistivities of the carbon/epoxy composite without SG are already lower than the lowest resistivities achieved with Kevlar/epoxy/SG. Thus, the volume resistivity is not expected to decrease, yet the surface resistivities are reduced by the effect of SG in the matrix and reach lower values than their Kevlar/epoxy/SG counterparts, probably due to dispersion of some carbon fiber fraction in the resin during impregnation of the plies.

### Composite materials prepared with carbon fabric/Kevlar combinations

Composite laminates were prepared from combinations of Kevlar and carbon fabric. Asymmetric layering provides the possibility to create composite materials with regulated surface resistivity for the different external laminates of the composite structure. Laminated composites were prepared in two different configurations:

1. asymmetric structure: (0/90 CF)_3_/(0/90 Kev)_3_,
2. symmetric structure: (0/90 CF)_2_/(0/90 Kev)_1_/(0/90 CF)_1_/(0/90 Kev)_1_/(0/90 CF)_2_.

The properties of the composite materials are presented in Table S3 and Table S4 (Supporting Information File 1). Laminated composites fabricated with the first configuration manifested increased DMA storage moduli, especially for the composition with epoxy/Triton X-15 which consistently showed higher moduli at all temperatures up to 120 °C (Table S3, Supporting Information File 1). Increased Young’s moduli were determined for all materials in a three-point-bend loading, while stress at yield was mostly unaffected. The *T*_g_ evaluated from the tan δ values of the DMA showed some scatter but remained at roughly the same level as the unmodified resin system.

The properties of laminated composite materials prepared with the second configuration are demonstrated in Table S4 (Supporting Information File 1). The introduction of SG leads to enhanced moduli, however, the various surface treatments do not further contribute to the moduli beyond these values. Interestingly, at the highest temperature of 120 °C, the relatively high values of DMA storage moduli are comparable with those of composites prepared with 10 ply CF/neat epoxy matrix. The enhancement in DMA storage moduli of composites with epoxy/SAA/SG binding matrices in comparison with the neat epoxy matrix seems to reflect the better wetting ability of the nanomodified matrix, which is expected to improve as temperature rises and is likely to explain the high moduli at 120 °C.

Surface resistivities of the asymmetric laminate in Table S3 demonstrate the ability to control one-sided conductivity or gradient conductivities of up to three orders of magnitude in a composite structure. Surface resistivities for both symmetric and asymmetric laminates are expectedly similar to the all-carbon laminates.

Table S4 indicates that, again, Kevlar plies interleaved between carbon plies do not prevent volume conductivity, especially with Triton X-15 and MEMO surfactants where resistivity is only one order of magnitude higher than the all-carbon laminates.

### Composite materials based on fiberglass fabrics

**Figure 3:**
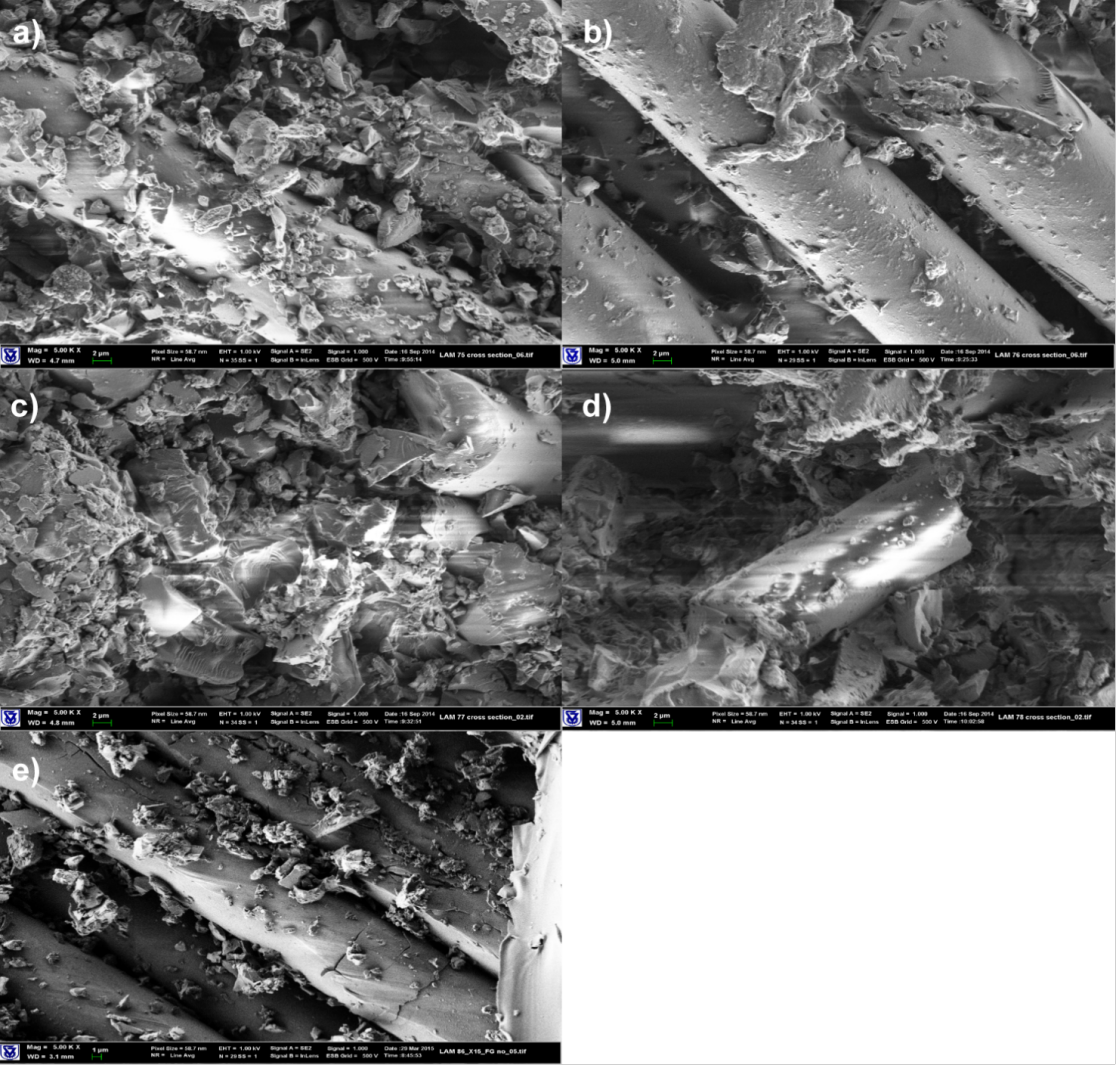
SEM images of composite materials based on FG fabrics (magnification: 5000×). (a) Neat Epoxy matrix, (b) epoxy/SG (2.5 pph) matrix, (c) epoxy/Triton X-100 (1 pph)/SG (2.5 pph) matrix, (d) epoxy/MEMO (1 pph)/SG (2.5 pph) matrix, (e) epoxy/Triton X-15 (1 pph)/SG (2.5 pph) matrix.

SEM images of FG/epoxy-laminated composites indicated no marked differences in the morphology of composite materials with the investigated SG modified matrices. The epoxy based compositions do not provide sufficient wetting and coverage of fiberglass filaments, even when surfactants were added (Figure 3).

The properties of laminated composite materials based on FG fabrics are summarized in Table S5 (Supporting Information File 1). As can be seen, significant improvements in electrical properties were achieved due to a percolation network being formed by the conductive matrix between the insulating fiberglass filaments. Surface and volume resistivities dropped down by 7–9 orders of magnitude. However, The thermo-mechanical properties of the composite materials indicated some deterioration. The DMA storage moduli slightly decreased, especially at elevated temperatures. Young’s moduli determined by 3-point bending remained at the level of the neat epoxy composites. A significant decrease in stress at yield values was observed. The reason for the latter deterioration could be attributed to the increased rigidity of the laminates expressed as lower strain at yield, or the incompatibility between the FG sizing and the various SAAs leading to agglomeration in the matrix phase, as shown in Figure 3.

**Figure 4:**
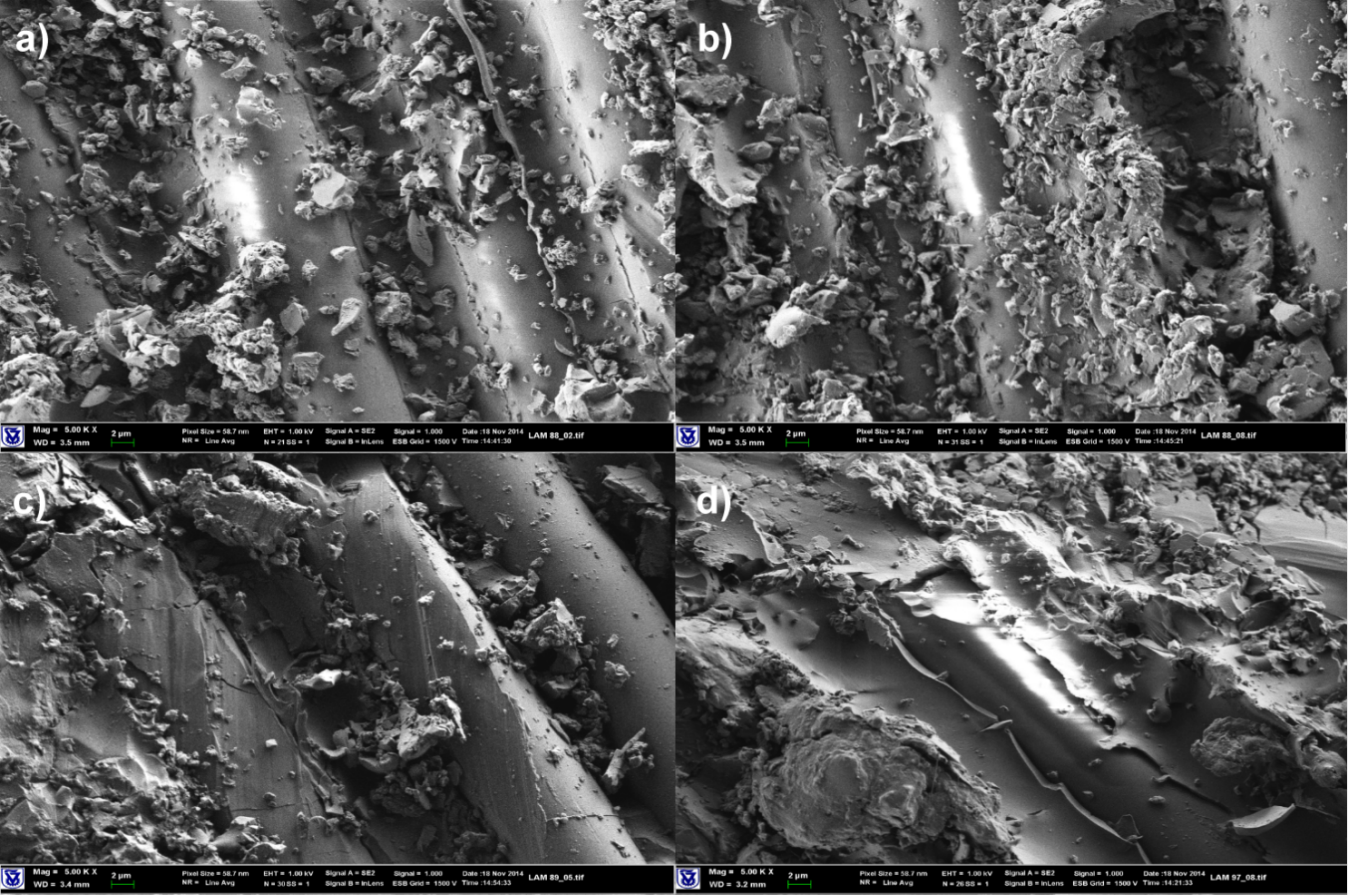
SEM images of composite materials based on FG fabrics, FG treated with MEMO or GLYMO (magnification: 5000×). (a) Epoxy/MEMO silane/SG (2.5 pph) matrix, FG treated with MEMO; (b) epoxy/MEMO SG (2.5 pph) matrix, FG treated with MEMO; (c) epoxy/SG (2.5 pph) matrix, FG treated with GLYMO; (d) epoxy/Triton X-100 (1 pph)/SG (2.5 ph) matrix, FG treated with GLYMO.

Experiments aimed at modification of the FG surface, using silanes with either acrylic or epoxy functionalities, in order to provide better wetting of filaments, were carried out, as explained in detail in the Experimental section. Examination of morphology and mechanical properties of laminated materials did not reveal any substantial changes in the performance of the laminates (Figure 4 and Table S5, Supporting Information File 1). It can be concluded that these types of silanes do not contribute to the improvement of FG/epoxy-based matrix compatibility.

Further FG treatments were carried out with (3-aminopropyl)triethoxysilane (AMEO), triethylenetetramine (TETA), and the non-ionic SAAs Triton X-100 and Triton X-15 dissolved in IPA at various concentrations. Results of electrical and thermo-mechanical tests are shown in Table S6 (Supporting Information File 1). As can be seen, modification of FG with AMEO led to deterioration of the properties of the laminated composite. After the addition of epoxy-functionalized silane GLYMO as a surfactant to the matrix composition the values of DMA storage moduli, Young's modulus and stress at yield were restored.

Interesting results were observed with the non-ionic hydrophobic SAA Triton X-15. Addition of this SAA to the epoxy/SG matrix blend, as well as the modification of FG with 1% solution of Triton X-15 led to improved mechanical properties. The SAA concentration must be balanced an excess of this SAA during FG modification (2% solution) had a negative effect on the stress at yield.

Improved thermo-mechanical properties of laminated FG composites were observed also with moderate concentrations of Triton X-100 applied for FG modification and incorporated into the matrix composition. Here, an excess of SAA in the FG fabric also results in a decrease of the stress at yield value. Increase in DMA storage moduli and stress at yield was noticed when FG was treated with TETA hardener. Further crosslinking at the interphase may take place when testing at high temperatures, explaining the higher modulus at 100 °C. Fine tuning of the epoxy composition at the interphase may turn out to be an interesting way to improve its properties, and there is some potential in this practice to improve properties.

**Figure 5:**
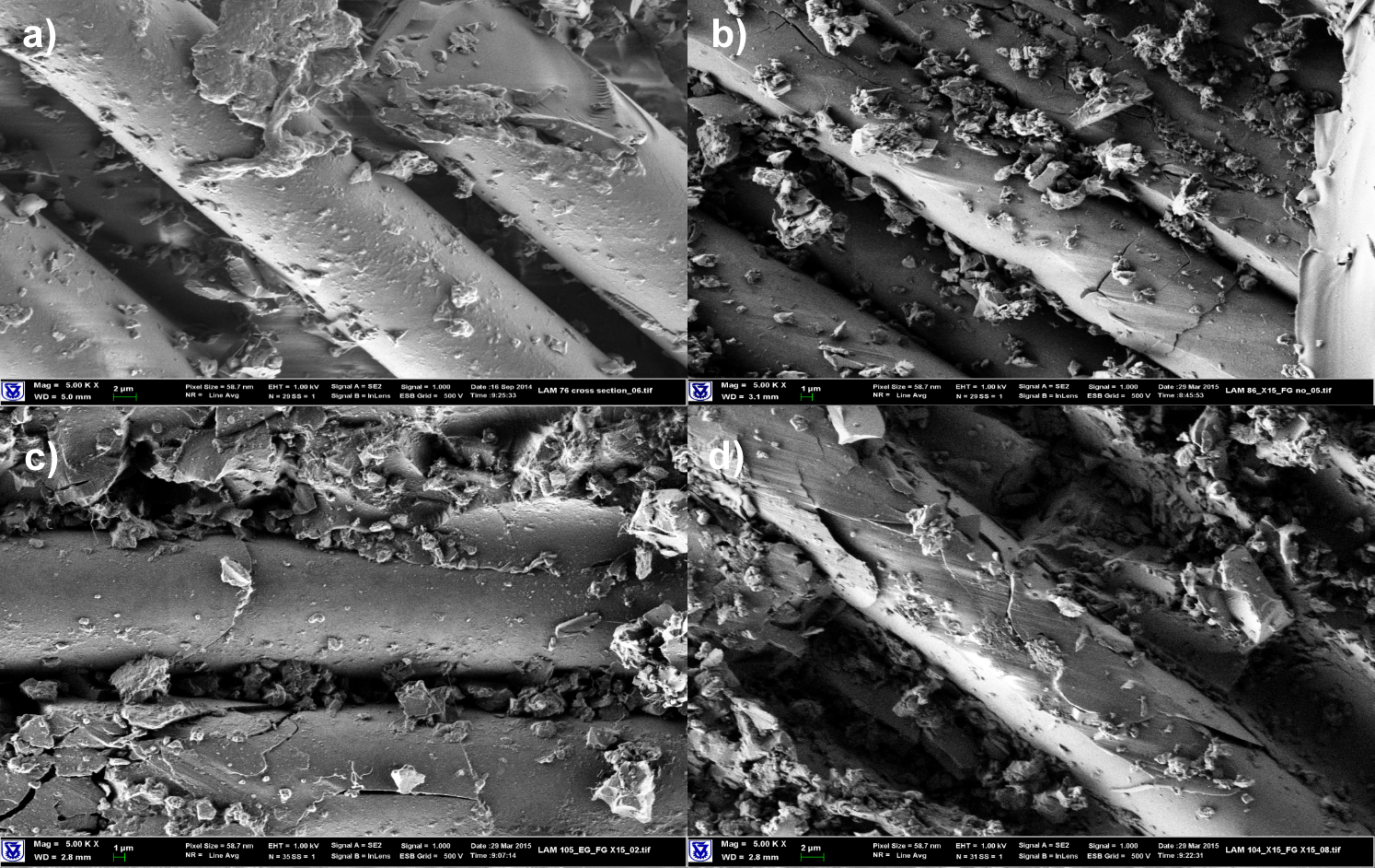
SEM images of composite materials based on FG fabrics, FG treated with 2% Triton X-15 (magnification: 5000×). (a) Epoxy/SG (2.5 pph) matrix; (b) epoxy/Triton X-15 (1 pph)/SG (2.5 pph) matrix; (c) epoxy/SG (2.5 pph) matrix, FG treated with 2% Triton X-15; (d) epoxy/Triton X-15 (1 pph)/SG (2.5 pph) matrix, FG treated with 2% Triton X-15.

**Figure 6:**
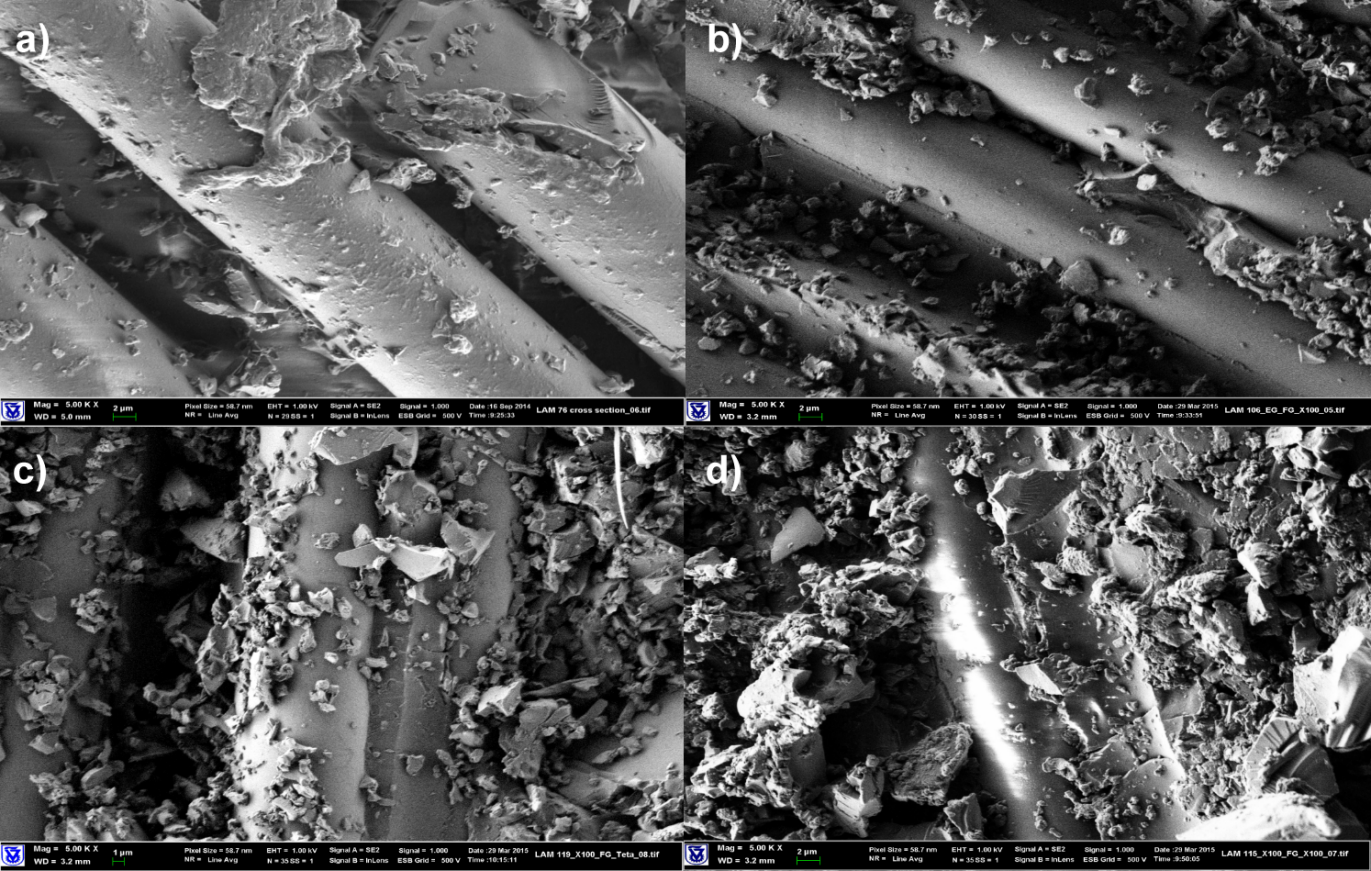
SEM images of composite materials based on FG fabrics, FG treated with Triton X-100 or TETA (magnification: 5000×). (a) Epoxy/SG (2.5 pph) matrix; (b) epoxy/SG (2.5 pph) matrix, FG treated with 2% Triton X-100; (c) epoxy/Triton X-100 (1 pph)/SG (2.5 pph) matrix, FG treated with 2% TETA; (d) epoxy/Triton X-100 (1 pph)/SG (2.5 pph) matrix, FG treated with 1% Triton X-100.

**Figure 7:**
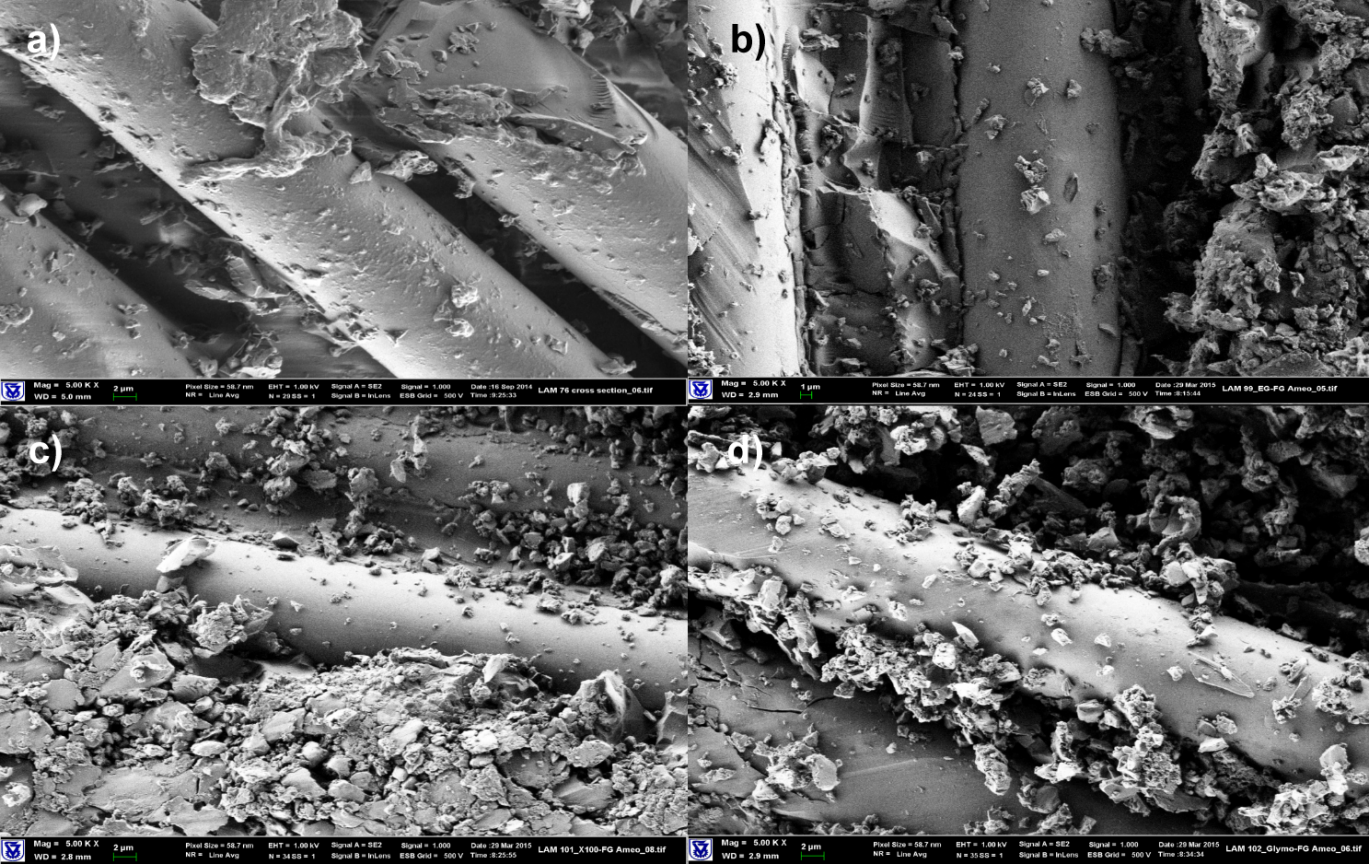
SEM images of FG fabric based composite materials, FG treated with 2% AMEO (magnification: 5000×). (a) Epoxy/SG (2.5 pph) matrix; (b) epoxy/SG (2.5 pph) matrix, FG treated with 2% AMEO; (c) epoxy/Triton X-100 (1 pph)/SG (2.5 pph) matrix, FG treated with 2% AMEO; (d) epoxy/GLYMO (1 pph)/SG (2.5 pph) matrix, FG treated with 2% AMEO.

SEM images of composite materials based on modified FG are shown in Figures 5–7. Morphology of the composite materials in Figure 5 explains the observed improvement in mechanical properties of epoxy/Triton X-15/SG laminated materials. The nanoparticle platelets cover the surface of FG fibers modified with Triton X-15. With Triton X-100 modification of FG, Figure 6, the coverage is less obvious since bare and decorated sections of FG filaments can be found. These results suggest a rather hydrophobic sizing has been applied to the FG, and this is reasonable since this sizing is recommended for epoxy compositions. The SEM image of laminates prepared with TETA modification of FG fibers in Figure 6 demonstrates a great number of small matrix fragments bonded to the surface of FG fiber. As can be seen, in composites based on GLYMO-modified epoxy matrix and AMEO-modified FG, more epoxy matrix fragments are bonded to the surface of FG filaments (Figure 7). The morphology of laminated FG composite materials depends on the chemical structure of FG surface layers and is finally reflected in the mechanical properties of the composites.

The unknown composition of the sizing material used for the FG filaments could be the reason for FG/matrix composition incompatibility. Hence, experiments were carried out to remove the original treatment in order to reveal an influence of selected FG impregnating material on the properties of laminated composites. Subsequently, composites based on heat-treated FG fabrics that were further modified with SAAs (procedure is described in the Experimental section) demonstrated higher DMA storage moduli, especially at elevated temperatures as shown in Table S7 (Supporting Information File 1). FG modified with Triton X-15, AMEO and TETA, and the epoxy/SG matrix modified with Triton X-15 and GLYMO manifested improved performance after removal of the commercial sizing.

## Conclusion

The exfoliation of commercially available expandable graphite (EG) is enhanced by impregnation in an epoxy/surfactant mixture, allowing on to downsize the nanoplatelets to "stacked" graphene (SG) or few graphene layers (FGL) platelets. The effect of such exfoliation on continuous-fiber-reinforced epoxy composites was studied in terms of electrical conductivity and thermo-mechanical properties at low loadings. Modification of epoxy resin by SAAs prior to EG impregnation enhanced intercalation of epoxy monomer between EG layers and further exfoliation. Morphology and compositions are reflected by the mechanical properties of composite laminates. Electrically conductive laminated composites, based on Kevlar or glass fibers, can be obtained by introducing treated stacked graphene at low concentrations.

The morphology of composite materials based on Kevlar fabrics varies depending on the composition of the matrix. Laminates prepared with neat epoxy or unmodified epoxy/SG exhibited bare surfaces of Kevlar fibers. Modification of epoxy resin with Triton X-100 and methacrylate-functionalized silane (MEMO) resulted in improved wetting of the Kevlar filaments with the nanocomposite matrix. Enhanced rigidity in three-point bending and increased DMA storage moduli were achieved for composites where EG was impregnated with epoxy resin modified by Triton X-100, Triton X-15 and MEMO. All Kevlar-based laminates demonstrated improved electrical properties, indicating the possibility to obtain electrically conductive composite materials on the basis of electrically insulating fibers using binder matrices with low content of inexpensive SG. Both surface and volume resistivity dropped by 7–8 orders of magnitude.

Laminated composites based on carbon fabric showed increased DMA storage moduli, improved thermal stability and increased Young's moduli. SEM images of SAA-modified epoxy/SG/carbon fabric composites revealed no distinctive effect of the epoxy composition on the morphology of laminates, yet the compounds demonstrated significant improvement in thermal stability at elevated temperatures, especially with a polyol surfactant. A reduction of electrical surface resistivity was registered, while volume resistivity remained at the level determined for the neat epoxy composite.

Composite materials consisting of Kevlar/carbon fabric combinations with epoxy/SAA/SG manifested increase in DMA storage moduli and enhanced Young's moduli. Asymmetric layering of the Kevlar/carbon layers provided the possibility to form hybrid composite materials with regulated surface resistivity for the different external plies of the laminate.

For fiberglass-based composites with various nanocomposite matrices no marked differences in the morphology were noticed. Epoxy-based compositions, even modified with SAA, do not provide sufficient wetting and coating of fiberglass filaments. DMA storage moduli and Young's moduli remained at the level of the neat epoxy matrix, or were even reduced slightly. Treatment of fiberglass fabrics using moderate concentrations (1–2%) of non-ionic SAA (Triton X-15 and Triton X-100), or TETA hardener in IPA allowed for improvement of mechanical and thermo-mechanical properties of FG composites. Electrical properties manifested significant improvement as surface and volume resistivities dropped by 7–9 orders magnitude.

Thermomechanical properties of FG-reinforced laminated composite materials can be improved by removal of the commercial sizing and modification of the fiberglass surface using moderate concentrations (about 1%) of either AMEO, non-ionic Triton X-15 or even TETA hardener. We suggest to develop strategies to fine-tune the crosslink density at the interphase. A graded crosslink density transition from the fiber to the matrix may prove beneficial.

## Supporting Information

Supporting Information contains all tables with the measured thermo-mechanical and electrical properties of all samples.

File 1 Thermo-mechanical and electrical properties.
