# Supplementary material for: Enhancement of mechanical and electrical properties of continuous-fiber-reinforced epoxy composites with stacked graphene
Source: Beilstein J Nanotechnol. 2017 Sep 12;8:1909–18. doi: 10.3762/bjnano.8.191 (PMC5629411; doi:10.3762/bjnano.8.191)
Supplement: File 1 — Thermo-mechanical and electrical properties. [file Beilstein_J_Nanotechnol-08-1909-s001.pdf]

Supporting Information

for

Enhancement of mechanical and electrical properties of continuous-fiber-reinforced epoxy composites with stacked graphene

Naum Naveh*[1,2], Olga Shepelev[2] and Samuel Kenig[1,2]

Address: [1]Shenkar College of Engineering and Design, 12 Anna Frank St., Ramat Gan 5252626, Israel and [2]Israel Plastics and Rubber Center, Technion City, Haifa 3200004, Israel

Email: Naum Naveh* - naumn@shenkar.ac.il

* Corresponding author

Thermo-mechanical and electrical properties

Table S1: Thermo-mechanical properties of composite materials based on Kevlar fabric.

SG, wt %	SAA	Fiber fraction in laminate, wt %	# plies	Electrical resistivity, conductivity		DMA Storage Modulus at temperature (°C), GPa					DMA	Flexural 3-point bending	
			Kevlar	Surface, Ω/sq	Volume, S/cm	25	50	75	100	120	T_g, °C	Stress at yield, MPa	Young Modulus, GPa
0	—	64.7	5	5.0e(14)	1.5e(15)	10.7	10.5	10.5	9.27	4.64	116.6	156	10.2
2.1	—	63.8	5	1.3e(8)	2.2e(8)	9.75	9.52	9.65	9.02	5.02	118.2	156	10.9
2.1	Polyol 4200	61.1	5	6.0e(7)	4.6e(9)	9.46	9.54	9.44	8.84	4.45	116.5	209	17.5
2.1	X-100	62.0	5	5.6e(6)	2.5e(7)	12.8	12.3	11.8	10.7	5.15	115.2	169	14.4
2.1	X-15	60.4	5	1.2e(9)	9.0e(9)	13.1	12.8	12.2	10.6	5.0	116	219	15.8
2.1	GLYMO	64.8	5	4.0e(5)	5.5e(6)	10.8	10.4	9.95	8.66	4.35	116.5	145	10.8
2.1	MEMO	64.9	5	2.0e(7)	3.0e(7)	13.0	12.5	11.6	9.06	4.58	116.2	168	11.3

Table S2: Thermo-mechanical properties of composite materials based on carbon-fabrics.

SG, wt %	SAA	Fiber fraction in laminate, wt %	# plies	Electrical resistivity, conductivity		DMA Storage Modulus at temperature (°C), GPa					DMA	Flexural 3-point bending	
			Carbon fabrics	Surface, Ω/sq	Volume, S/cm	25	50	75	100	120	T_g, °C	Stress at yield, MPa	Young Modulus, GPa
0	—	56.1	10	9e(6)<br>3.5e(12)	3.4e(5)	39.4	38.6	38.1	31.5	11.2	111.7	681	44.5
2.1	—	57.9	10	1.1e(4)<br>7.5e(4)	6.5e(5)	42.9	42.0	41.5	34.4	16.4	109.5	624	46.0
2.1	Polyol 4200	59.6	10	1e(4)<br>7e(4)	3.3e(5)	45.7	44.9	44.1	39.5	20.5	111.7	690	51.1
2.1	X-100	58.7	10	1e(4)<br>2e(5)	1.3e(5)	43.1	42.6	41.7	32.7	17.3	108.3	669	50.1
2.1	X-15	56.8	10	8.5e(3)<br>2.8e(5)	2.5e(5)	41.1	40.8	40.4	37.4	14.9	113.2	672	47.7
2.1	GLYMO	58.3	10	1.2e(4)<br>2.3e(5)	1.9e(5)	42.4	41.6	41.3	32.7	17.1	108.9	631	49.5
2.1	MEMO	58.7	10	1.6e(4)<br>6.3e(4)	2.8e(5)	43.8	43.2	42.4	34.1	18.0	109.2	669	48.9

Table S3: Thermo-mechanical properties of asymmetric composite materials based on Kevlar/carbon fabrics.

Layering: $(0/90\ CF)_3/(0/90\ Kev)_3$.

SG, wt %	SAA	Fiber fraction in the laminate, wt %	# plies	Electrical resistivity, conductivity		DMA Storage Modulus at temperature (°C), GPa					DMA	Flexural 3-point bending	
			Kevlar/ Carbon	Surface, Ω/sq	Volume, S/cm	25	50	75	100	120	T_g, °C	Stress at yield, MPa	Young Modulus, GPa
0	—	61.0	3 / 3	8e(13)<br>1.5e(5)	6.5e(9)	11.7	11.4	11.7	10.8	5.02	117.1	295	15.7
2.1	—	57.3	3 / 3	3.5e(8)<br>1.5e(4)	1.0e(9)	11.1	11.8	12.9	11.9	6.74	111.4	288	19.1
2.1	Polyol 4200	59.6	3 / 3	5e(8)<br>4.7e(4)	1.4e(9)	12.3	12.5	13.7	12.1	6.87	108.1	311	21.6
2.1	X-100	59.9	3 / 3	7.3e(7)<br>6.5e(4)	1.0e(8)	11.2	11.6	12.4	11.2	5.60	112.4	283	21.5
2.1	X-15	58.9	3 / 3	7.7e(7)<br>5.0e(4)	6.5e(8)	16.1	15.4	15.7	14.6	9.00	112.6	289	17.9
2.1	GLYMO	57.9	3 / 3	1.5e(7)<br>3.5e(4)	1.1e(8)	11.4	11.8	12.0	11.2	5.58	114.9	282	21.4
2.1	MEMO	59.5	3 / 3	2.1e(9)<br>6.0e(4)	1.5e(9)	16.2	16.0	15.8	13.8	7.27	115.2	302	21.6

Table S4: Thermo-mechanical properties of symmetric Kevlar/carbon fabric based composite materials.

Layering: $(0/90\ CF)_2/(0/90\ Kev)_1/(0/90\ CF)_1/(0/90\ Kev)_1/(0/90\ CF)_2$.

SG, wt %	SAA	Fiber fraction in the laminate, wt%	# plies	Electrical resistivity, conductivity		DMA Storage Moduli at temperature (°C), GPa					DMA	Flexural 3-point bending	
			Kevlar/ Carbon	Surface, Ω/sq	Volume, S/cm	25	50	75	100	120	T_g, °C	Stress at yield, MPa	Young Modulus, GPa
0	—	62.3	2 / 5	2.9e(4)	5.7e(9)	29.2	29.5	31.9	31.1	17.3	115.7	474	38.2
2.1	—	61.2	2 / 5	1.3e(4)	3.0e(8)	35.2	34.8	34.4	31.5	20.2	115.9	433	38.8
2.1	Polyol 4200	58.5	2 / 5	1.3e(4)	3.2e(7)	36.0	35.5	34.8	30.3	16.6	113.5	439	36.3
2.1	X-100	57.2	2 / 5	1.5e(4)	1.4e(7)	33.6	32.7	32.4	27.6	14.0	111.6	418	35.6
2.1	X-15	57.9	2 / 5	2.7e(4)	2.6e(6)	33.5	33.2	33.1	29.8	14.6	114	401	35.8
2.1	MEMO	58.3	2 / 5	3.3e(4)	3.9e(6)	34.5	33.7	33.1	30.2	17.3	113.1	427	37.7

Table S5: Thermo-mechanical properties of composite materials based on fiberglass fabrics.

SG, wt %	SAA type in the matrix blend	Fiber fraction in the laminate, wt %	# plies	Electrical resistivity, conductivity		DMA Storage Modulus at temperature, °C, GPa					DMA	Flexural 3-point bending	
				Surface, Ω/sq	Volume, S/cm	25	50	75	100	120	T_g, °C	Stress at yield, MPa	Young Modulus, GPa
0	—	63.7	10	3.5e(14)	2.8e(14)	18.6	17.9	16.6	13.6	6.81	112.2	562	18.6
2.1	—	62.9	10	1.2e(6)	1.5e(6)	18.6	17.8	16.6	13.8	7.08	112.1	456	18.9
2.1	Polyol 4200	63.8	10	3.6e(5)	1.2e(6)	18.4	17.7	16.6	12.4	6.27	107.2	479	18.7
2.1	X-100	62.6	10	1.6e(6)	5.5e(6)	19.1	18.3	16.8	11.9	7.31	105.6	441	18.2
2.1	X-15	61.1	10	5.2e(5)	1.7e(6)	16.5	15.6	14.3	10.4	5.69	106.3	393	16.9
2.1	GLYMO	62.9	10	1.2e(5)	1.7e(6)	17.5	16.5	15.2	11.4	6.53	107.7	455	17.9
2.1	MEMO	64.8	10	3.0e(6)	9.1e(6)	18.5	17.8	16.6	12.9	7.42	107.8	471	18.7

Table S6: Thermo-mechanical properties of composite materials based on fiberglass fabrics.

FG modified. Matrix composition: Epoxy DER 331 -100; TETA -14.1; SG 3772 (600 °C for 15 min) – 2.5 pph; SAA – variable.

SG, wt %	SAA in the matrix blend, type/ %	FG treatment, type/ %	Fiber fraction in the laminate, wt %	# plies	Electrical resistivity, conductivity		DMA Storage Modulus at temperature (°C), GPa					DMA T_g, °C	Flexural 3-point bending	
					Surface, Ω/sq	Volume, S/cm	25	50	75	100	120		Stress at yield, MPa	Young Modulus, GPa
0	-	-	63.7	10	3.5e(14)	2.8e(14)	18.6	17.9	16.6	13.6	6.81	112.2	562	18.6
2.1	-	-	62.9	10	2.5e(6)	1.5e(6)	18.6	17.8	16.6	13.8	7.08	112.1	456	18.9
2.1	MEMO	MEMO	62.7	10	4.0e(7)	2.5e(7)	17.6	17.0	16.0	12.2	6.67	107.8		
2.1	X-100	GLYMO	64.3	10	6.0e(6)	4.9e(6)	17.1	17.2	17.0	15.1	7.51	117.3		
2.1	-	AMEO/ 2	60.5	9	1.7e(7)	1.2e(8)	15.6	14.7	13.4	10.9	5.19	114.2	412	18.6
2.1	GLYMO/ 1	AMEO/ 2	60.2	9	6.2e(6)	2.0e(7)	18.5	17.7	16.5	14.4	6.79	114.2	553	20.6
2.1	X-100/ 1	AMEO/ 2	59.8	9	3.8e(6)	2.8e(6)	16.5	15.9	14.8	12.5	5.49	113.3	522	19.3
2.1	X-15/ 0.5	-	64.4	10	3.5e(7)	5.0e(7)	19.1	18.5	17.8	15.0	6.94	112.1	600	21.1
2.1	-	X-15/ 2	64.3	9	5.2e(6)	1.8e(7)	17.9	17.3	16.7	14.5	8.03	114.1	465	19.0
2.1	X-15/ 2	X-15/ 1	63.9	9	3.5e(6)	1.9e(7)	19.2	18.6	17.9	15.5	7.58	113.6	579	20.3
2.1	-	X-100/ 2	65.0	9	1.1e(6)	1.2e(6)	18.7	18.1	17.7	15.1	7.78	113.8	564	20.0
2.1	X-100/ 0.5	X-100/ 1	63.1	9	4.0e(7)	4.1e(7)	20.1	19.5	18.7	15.6	7.36	113.0	553	19.1
2.1	X-100/ 1	X-100/ 2	63.9	9	1.1e(6)	1.2e(6)	18.2	17.5	16.3	12.6	6.83	110.1	419	18.8
2.1	X-100/ 0.5	TETA/ 2	64.5	9	8.0e(6)	1.2e(7)	20.3	19.7		17.7		-	559	19.4

Table S7: Effect of FG heat treatment.

SG, wt%	SAA in the matrix blend, type/%	FG heat treatment at 600°C/2 hrs	FG surface treatment, SAA type/%	DMA Storage Modulus at temperature (°C), GPa			
				25	50	75	100
0	NONE	NO	NONE	18.6	17.9	16.6	13.6
0	NONE	YES	NONE	18.1	17.5	16.9	16.3
2.1	X-15/0.5	NO	X-15/1	18.0	17.3	16.5	13.6
2.1	X-15/0.5	YES	X-15/1	18.9	18.2	17.5	16.8
2.1	GLYMO/1	NO	AMEO/1	17.1	16.4	15.4	14.2
2.1	GLYMO/1	YES	AMEO/1	17.7	17.1	16.5	15.8
2.1	GLYMO/1	YES	TETA/1	18.9	18.2	17.6	17.0
